# Supplementary material for: Patterns of Intron Gain and Loss in Fungi
Source: PLoS Biol. 2004 Nov 30;2(12):e422. doi: 10.1371/journal.pbio.0020422 (PMC532390; doi:10.1371/journal.pbio.0020422)
Supplement: Table S1 — Also available at http://genes.mit.edu/NielsenEtAl/. (4.3 MB ZIP). [file pbio.0020422.st001.zip › NielsenEtAl/html/1308.html]

AN6894.1.NCU00181.1.MG06417.1.FG05403.1


```
 CLUSTAL W (1.82) Multiple Sequence Alignments - Introns Inserted


Sequence 1: NCU00181.1	597 aa
Sequence 2: FG05403.1	588 aa
Sequence 3: MG06417.1	563 aa
Sequence 4: AN6894.1	580 aa
Alignment Length: 628 aa
Number Identitical Residues: 249 aa
Alignment Score (without introns) 12510


MG06417.1 	MGKG~TDKLY~ITHSEWASSDSFSASSGARD-----RPGEGRFKKLPFNFCAASLQPFKN
NCU00181.1	MGKG~TDKLY~ITHSEWSSSDAYGASTGANAG-ARAQRRGASFKKLPFNFCAASLQPFKN
FG05403.1 	MGKG~TDKLY0ITHSEWSSADAFSPSIGAGA--SRNQQATASFRRLPFNFCAASLQPFKN
AN6894.1  	MGKG1TDKLY0ITHSEWASEDAYSASAGAGVGKARRGVGEASFKRLPFRFCSLSLQPFEH
          	**** ***** ******:* *::..* **  ..:      . *::***.**: *****::

MG06417.1 	PVCTSDGTIFDVEVISAWLEKS-KTNPVDGEPLAARDLIKLNFARN------------AQ
NCU00181.1	PVCTPDGTIFDVEVIGSWLEKH-KTNPVTGEPLSAKDLIKLNFARNGDTTDS----DENK
FG05403.1 	PVCTPDGTIFDVEVIGVWLEKHPNQNPVTGEPLQKKDLIRLNFARNSESDSLGAGLSDGK
AN6894.1  	PVCTQSGTIFDLTSILPWIKKH-GTNPVDGTPLKGSDLIKLTIAKN------------DA
          	**** .*****:  *  *::*    *** * **   ***:*.:*:*              

MG06417.1 	DDLIDPVSYKVFTDNTHIVAVRHGAYANVFTWDTVERMNVKAKMWRDLVDDAEFGRADII
NCU00181.1	GDLIDPVTFKVFTDNTHIVAIRHGSYANVFAWETVERMNIKPKMWRDLVDDEEFGRRDII
FG05403.1 	GDLIDPVTYKVFTDNTHIVAIRHGTYANVFAWDTVDRMNIKAKSWRDLVDDEEFTRADII
AN6894.1  	GEYIDPVTYKVLTDNTHVVALRN--TGNVFAWDTIERLNIKGKMWRDLVSDEEFTRKDII
          	.: ****::**:*****:**:*:   .***:*:*::*:*:* * *****.* ** * ***

MG06417.1 	TLQDPQN-AAARDLSRFRHLKDGGEALQTREQEEERK--AGNVNVNALGRIGEKVLRAKE
NCU00181.1	TLQDPQNVSASRDLSQFKYLQDGQDAILTKEQEEERK--GGTVNIEALGRVGEKVLRAKE
FG05403.1 	TLQDPQN-AASRDLNQFKFLKEGHEAQLTKEQEEERN--AGNINAGALGSMGEKVSRAKA
AN6894.1  	TLQDPQN-IESRDLSRFNHIKEG-ESGLSDEQLREREDPSRNVNVNALGSS-AKILKARE
          	*******   :***.:*..:::* ::  : ** .**:... .:*  ***    *: :*: 

MG06417.1 	AVEKAREA----GVDVNSTTKSQALLAKASGAAN------GRQAAQSHAARKLPANAAAY
NCU00181.1	AVERARAARQAGGADVNRL--TQALTTTSTNSATNNNKTAIARGQSLIQERKRPANAATY
FG05403.1 	AVEKARKAREQGG-DVNRS--STALTKP-TGAGT------VVR-QSMINDKKLAVNSATY
AN6894.1  	AVAKARQERAQKAGNAAST--PVAKTDAPVKSAQ--------KTASYQSGKPVPYNAARY
          	** :**      . :.     . *    .  :.         :  .    :  . *:* *

MG06417.1 	TTGKAAASFTSTSLTPEKSGERALLSNEEWMLKP-KRVKNQGYARIETNSGDLTIELHTD
NCU00181.1	TTGLTAASFTSTGLTPSTSGSLALLSDEQYLLKPSHRIKNKGYVRMETNLGPLTLELLPE
FG05403.1 	TTGKAAASFTSTGLTPETSGERALLSDEEYMLKP-KRVKATGFARMETNMGDLTIELYPE
AN6894.1  	TTGLAAASFTSTGMTPHTSAELALLSDEDYMLKR-GRVKQKGYARISTTSGDINLELHTE
          	*** :*******.:** .*.. ****:*:::**   *:*  *:.*:.*. * :.:** .:

MG06417.1 	TAPKAVWNFVKLAQRGYYDGITFHRNIRNFMIQGGDPTGTGRGGSSIWGKNFEDEFDGPR
NCU00181.1	FAPKAVWNFLRLSEKGYYRDVAFHRSIRNFMIQGGDPSGTGRGGSSIWGKNFEDEFEGPN
FG05403.1 	FAPKAVWNFIKLSQTGYYKGVAFHRNIPNFMIQGGDPSGSGRGGQSVWGKYFDDEFDGPM
AN6894.1  	YAPKAVWNFIKLAKKGYYKDVTFHRNIKGFMIQGGDPTGTGRGGESIWGKYFNDEFEGPL
          	 ********::*:: *** .::***.* .********:*:****.*:*** *:***:** 

MG06417.1 	THNARGIM~SMANKGKNTNSSQF~FITYRATKHLDRKHTIFGRVVEGLD----VLDKLED
NCU00181.1	THSARGIV~SMANKGKNTNSSQF~FITYRPASHLDRKHTIFAKVIEGQDT---TLTAMEN
FG05403.1 	THNGRGTL1SMANKGKNTNSSQF~FFAYKPTPHLDRKHTVFGKVVENIN----VLSKMEN
AN6894.1  	KHDSRGTL~SMANKGKNTNSSQF2FIAYRALPHLNLKHTIFGHVIDDPTPSSPTLNKLET
          	.*..** : ************** *::*:.  **: ***:*.:*::.  .::..*  :* 

MG06417.1 	IPTD-GSDRPLHKIAINKVTVFIDPFEEFLREKGQKEEAEA----QKAEEQRHGG--SYD
NCU00181.1	VATD-GSDRPLNKIVIKDMIILIDPFAEWMKEKKQKEGEEE----RKREVARQGG--TED
FG05403.1 	VPTD-GSNRPLNKILIKDIVILLDPFAEFQKQKQANELQTK----EREKIRLQGG--TDD
AN6894.1  	HPVNPTTNRPTPDIRIVDVTIFVDPFEEFLKQKKQSEQSAENEANRTAENDEEGSRRAED
          	 ..:. ::**  .* * .: :::*** *: ::*  .*    ..:..  :   .*.  : *

MG06417.1 	DHVTWTGKRIRGD---NGQALSNDTN----VGKYLNI----EATTKDSSAPDDGAGLEAE
NCU00181.1	DRTTWTGKRIRADGTMEGQGMGEGGGGGPKVGKYLDVGAVKKAATTTTTTTRKAEEEEVD
FG05403.1 	DKTTWTGKRIRSDGSMENTGAGES------VGKYLKT-----TTQQSTPTVNEADLEDVD
AN6894.1  	DQITWTGKRVRGP----GASKEESST---TVGKYLKAA---LSERAAHDEDEIVEFVDEE
          	*: ******:*.     . .  :.     .*****. .    :              : :

MG06417.1 	DWEQPARKKMK-PGGFGNFDSW~----------
NCU00181.1	TWEEPVRKKAK-MGGFGNFDGW~----------
FG05403.1 	TWEEPVRKKAK-GGGFGNFDNC2ITPMAYDDFN
AN6894.1  	PTSEPAKKKFKGIGGFGDFSSW~D---------
          	  .:*.:** *. ****:*..
```
